# Analysis on Influences of College Students’ Psychological Capital in Entrepreneurial Learning Engagement

**DOI:** 10.3389/fpsyg.2020.02029

**Published:** 2020-08-18

**Authors:** Baoge Zhang, Qiuyan Xu, Song Han, Lan Jiao

**Affiliations:** ^1^Faculty of Teacher Education, Ningbo University, Ningbo, China; ^2^Department and Institute of Psychology, Ningbo University, Ningbo, China; ^3^Faculty of Education, Northeast Normal University, Changchun, China

**Keywords:** PC, ELE, PE, structural equation model, mediation effect

## Abstract

College students’ entrepreneurial learning engagement (ELE) is a key link that affects the success of future entrepreneurship. To analyze the influencing factors of the psychological capital (PC) dimension in college students’ ELE, a total of 211 college students were selected from colleges in the Ningbo area for questionnaire survey. The principal component analysis method was used to test the difference validity of PC and ELE. One-way analysis of variance was used to analyze differences in demographic variables between PC, ELE, and positive emotions (PEs). Besides, the structural equation model was used to analyze the mediating role of PEs in PC and ELE. In addition, there was a significant difference between the unrestricted model of PC–ELE and the restricted model (*p* < 0.05), and the difference between the potential dimensions of “mental capital-learning input” was generally satisfactory; there were significant differences at the professional level (*p* < 0.01); PC had significant differences in family economic status (*p* < 0.01); the indirect path coefficients of PE added to the relationship between PC and ELE were 0.106 and 0.211, respectively, and there was no significance (*p* > 0.05). In short, the PC of college students has a significant positive influence (PI) on ELE, PC differs significantly in family economic status, and PEs differ significantly at the professional level. The research results show that there is no mediating effect of PEs in the relationship between PC and college students’ ELE.

## Introduction

Entrepreneurship is a systematic project closely related to the society and people. It creates some new products and services that society needs. To succeed in entrepreneurship, the entrepreneur’s organizational ability and social communication ability should be improved (Cadenas et al., 2020). However, college student entrepreneurs mainly make decisions on an impulsive basis, without deliberate consideration, systematic investment in learning, and market research and analysis. This makes college student entrepreneurs lack the comprehensive quality and necessary professional skills for entrepreneurship to overcome all kinds of difficulties, resulting in an eventual failure (Ye, 2020; Zhang et al., 2020). Therefore, it is very necessary to invest in entrepreneurial engineering. University students need to be realistic, have a passion for entrepreneurship, and develop the ability to engage in continuous learning (Jena, 2020). For example, individual skills such as acquiring knowledge related to entrepreneurial theory, cultivating social communication skills, improving team management, enhancing the ability to respond to emergencies, learning self-cognition and scientific planning are the basic necessary qualities that an entrepreneur must possess. In addition, many colleges and universities also have actively carried out entrepreneurship education courses for college students in accordance with the development of the times and thus provide assistance for college students’ entrepreneurship learning (Al-Ani et al., 2020). In short, entrepreneurial learning engagement (ELE) is the foundation of entrepreneurial success, and college students’ ELE influences the future development of entrepreneurship greatly. Psychological capital (PC) refers to a positive psychological state that individuals show during their growth and development, which surpasses human and social capitals and advances personal improvement (Trung et al., 2020; Younis et al., 2020). The factors influencing ELE include internal factors and external factors; internal factors are mainly characteristic variables, which are usually difficult to change (Hu and Yuan, 2020). With increasing researches on positive psychology and behavior, the construction of positive behavior standards (PC), which conforms to development-, measurement-, and performance-oriented characteristics, begins to receive extensive attention (Bazan et al., 2020). Entrepreneurship learning is not all smooth sailing, and due to differences in individual character and psychological quality, college students have different levels of acceptance toward entrepreneurship education in colleges (Liu and Chen, 2018).

In the actual learning process, it is necessary to fully understand and explore the inherent positive psychological qualities of individuals. The success of ELE is related to the PC of college students to some extent (Stefanic et al., 2020). Therefore, this study considered exploring the influencing factors of college students’ ELE from the perspective of PC. The reasons for the low ELE may be the attitude toward learning. PEs refer to people’s feelings of pleasure, and they have a guiding effect on individual cognition.

## Literature Review

### Research Background

#### Research on the Psychological Effect of Entrepreneurs

The creation of new businesses is not a key indicator of entrepreneurial success, and the sustainability of entrepreneurs is largely determined by positive collective psychology. The study of Tang (2020) showed that positive PC could promote the sustainability of entrepreneurs and enable them to successfully establish new enterprises. In addition to PC, some researchers pointed out that family entrepreneurial enterprises may also affect the entrepreneurial motivation of entrepreneurs. However, Lee et al. (2019) analyzed the entrepreneurial motivation of family and nonfamily businesses based on psychological ownership and found that there was no difference in the importance attached to entrepreneurship between the two. Based on the national strategy of “mass entrepreneurship and innovation,” entrepreneurship education has become an exploration field in all sectors of Chinese society, especially in universities, and students are the new major groups of entrepreneurs. However, for college students who have just entered the society, the psychological effect will have a significant effect on entrepreneurship. Laudano et al. (2019) explored the influence of female college students’ entrepreneurial intention on their psychology, environment, and personality traits. The results showed that the individual’s attitude, intention, and other psychological factors could affect the entrepreneurial attitude of female college students, and the tendency to take risks was an important prerequisite for entrepreneurial attitude (Feng and Chen, 2020). Carol (2019) explored the correlation between college students’ happiness and entrepreneurship and found that entrepreneurial experience of college students could interact in the pursuit of self-happiness, indicating that entrepreneurship of college students was inseparable from their pursuit of happiness. To sum up, the success or failure of entrepreneurs is largely influenced by the psychological effect of entrepreneurs, but the internal mechanism of the influence of entrepreneurial psychology on college students is still unclear (Siu et al., 2013).

#### Relative Studies About ELE

ELE is a concept proposed for those who are working, and Feldman et al. (2014) defined it as the motivation for work and the degree of their participation in work. Participation in work refers to the enthusiasm for work, pleasure, and other psychological states. When a self-employed person is sufficiently engaged, he will put all his energy into his role and fully present himself. However, when a self-employed person is not engaged in his work, he will separate himself from his work role and eventually give up his position. So far, many experts have conducted related research on ELE. The study of Vanno et al. (2014) showed that ELE was closely related to job performance. The study of Dada and Fogg (2016) showed that entrepreneurship orientation had a positive effect on the organizational learning of small- and medium-sized enterprise, and that the participation of enterprises/universities was an important factor to promote the entrepreneurship orientation of college students. Boso et al. (2018) proved that the entrepreneurial failure experience would affect the performance of the enterprise, but improving the enterprise’s learning level would make the entrepreneur more alert when new business opportunities arise, which suggested that in the process of starting a business, it was necessary to constantly improve the learning level of the enterprise, so as to avoid as much as possible the effect of entrepreneurial failure on the performance of the new enterprise. To sum up, showing a positive state of learning during the entrepreneurial process can provide the enterprise’s job performance through continuous progress. Can the positive entrepreneurial learning attitude stimulate the entrepreneurial passion of college students and then affect the performance of college students’ entrepreneurship? These remain to be proven.

#### Research Status of the Relationship Between PC and ELE and Between PEs and PC

Employees with high PC are more likely to be satisfied with their jobs. Accordingly, PC has a significant positive influence (PI) on work performance, which has been proved in previous studies. It is believed that PC is related to students’ academic performance at the individual level (Luthans et al., 2012), just like ELE, and PC has a PI on students’ ELE. PE refers to people’s feelings of pleasure. PEs have priming and expanding effects on cognition and can build individual resources (Guo and Wang, 2007). The theory of the expansion and construction of the PEs of Fredrickson (the broaden-and-build theory of PEs) holds that PEs can expand the scope of individual cognition. In experiment tasks, individuals with PEs exhibit higher creativity, solve problems faster, and make more detailed and careful decisions. Therefore, it can be inferred that individuals with PEs have higher ELE (Rogoza et al., 2018).

### Theoretical Significance and Practical Significance

Recently, PC has been studied by many researchers, but most of the empirical studies are concentrated on the enterprise. It was found that PC had a PI on job performance. From 2014, college students have been taken as the research objects to explore the relationship between PC and learning performance. The study found that PC can promote college students’ learning performance. In China, many college students do not have a goal in school, consider college a place for relaxation, and do not devote themselves to learning. Only when they are employed do they find that they lack professional qualities. It is found that college students’ ELE can be improved by developing the PC of college students. To push students to learn, the institutions of higher education can bring up the students’ PC into the consideration category and then improve the students’ ELE to achieve the purpose of cultivating talents (Liu et al., 2019).

In summary, most of the current research from the perspective of PC focuses on the direction of enterprise employees, and there were few analyses involving college students’ ELE. Based on this, a total of 211 college students were selected in Ningbo area colleges and universities to conduct a questionnaire survey, and the verification tests and structural equation models were used to analyze the mechanism of PC’s effect on college students’ ELE, so as to help college students improve ELE.

## Materials and Methods

### Research Subjects

The subjects in this study were students from Ningbo University. The authors conducted the experiment through the questionnaires, gave out 300 questionnaires, and recovered 211 valid questionnaires. The valid recovery rate was 70.3%. Among the subjects, there were 56 male subjects, accounting for 26.5% of the total; there were 155 female subjects, accounting for 73.5%. In addition, 100 subjects major in psychology, accounting for 47%; 36 subjects major in physical education major, accounting for 17%; 34 subjects major in primary education, accounting for 16%; 14 subjects major in educational technology, accounting for 6%; and 27 subjects major in preschool education, accounting for 11%. These were students from families with a monthly income varying from 1,000 to 6,000.

### Experimental Hypotheses

The experimental hypotheses in this research are as follows: (1) PC has a predictive effect on learning involvement. (2) PE serves as the intervening variable in the mechanism in which PC has an effect on learning involvement.

### Variable Measurement

For the PC variable, the authors used PC questionnaires (Minglu et al., 2020) adapted by Zhang Kuo, which included 26 measurement subjects to be used to measure this variable. This scale has made its four elements based on the published and extensively recognizable standardized scale with its reliability and validity verified according to the practical situation of Chinese university students. This scale included self-efficiency, hope, resilience, and optimism. The authors used the Likert-6 scoring method, which utilized the numbers 1–6 to represent the different attitudes of the subjects from dissatisfaction to satisfaction. The subjects included the judgments such as “many admire my capacity,” “my opinions and competences are unordinary,” and “I am very confident about my capacity.” The consequence of confirmatory factor analysis (CFA) demonstrated that the data conformed to the model [χ^2^ = 577.032, *df* = 293, root mean square error of approximation (RMSEA) = 0.068, goodness of fit index (GFI) = 0.823, normed fit index (NFI) = 0.839, Tucker–Lewis index (TLI) = 0.883]. The internal consistency α coefficients of the four dimensions were 0.861, 0.808, 0.792, and 0.868. The overall internal consistency α coefficient of this scale was 0.864. According to the previous researches, the average value of the four dimensions was used as the measured value.

For the PE variable, the Positive and Negative Affect Schedule (PANAS) revised by Qiu Xue in this research (Dunn et al., 2020) was adopted, and the original one was compiled by Watson. In the revised version, there were 18 adjectives concerning emotion. This scale concerned two dimensions – positive emotion and negative emotion. A total of nine adjectives were related to the PE involving active, enthusiastic, happy, and excited subjects. This research only utilized the PE scale and used the Likert-5 scoring method, and the numbers 1–5 indicate different PEs with the degree varied from very little to very high. Furthermore, its internal consistency α coefficient was 0.92.

For the ELE variable, the authors used the 17 subjects in the Utrecht Work Engagement Scale (UWES) to measure the students’ ELE (Setyaji et al., 2020) and used the Likert-5 scoring method with the numbers from 1 to 5 demonstrating different degrees of conformity from “very inconsistent” to “very consistent.” This scale involved subjects such as “I am willing to study since I get up in the morning” and “I feel energetic in the learning process.” The internal consistency α coefficient of this scale was 0.92.

For the control variable, personal characteristics that might influence students’ PC, ELE, and PEs were controlled, including gender, specialization, grade, and family monthly income. Among them, gender, specialization, and grade were classified variables, and monthly family income was a continuous variable.

### Statistical Analysis

This research used the software SPSS 16.0 and the software AMOS 21.0 to conduct the data analysis. The analysis item included (1) conducting the descriptive statistics and correlation analysis; (2) examining and investigating the reliability and validity of the questionnaires through the validity analysis and the CFA; (3) constructing structural equation through AMOS to investigate the correlation among the PE, PC, and learning involvement; (4) using principal component analysis to test the difference validity of PC and ELE; and (5) using one-way ANOVA to analyze the differences of PC, ELE, and PEs among demographic variables.

## Experimental Results

### Validation Test of Variable Discriminant Validity

Firstly, this study carried out CFA to test the difference validity of two variables, PC and ELE, and used a single group to generate two models, which were the unrestricted and restricted models (Kurihara et al., 2020). Then, the differences in the chi square values of the two models were compared. If the chi square value difference was larger and reached a significant level (*p* < 0.05), there was a significant difference between the two models. The smaller the chi square value of the unrestricted model, the lower the correlation between latent trait (factor dimension). When testing the survey data and the matching degree of the model, the average number of topics of four dimensions of PC was first calculated, and then the average number of the four dimensions was used as the four measurement indicators of PC. In the same way, the average number of topics of three dimensions of ELE was calculated, and then it was used as the three measurement indicators of ELE, as shown in Tables 1, 2.

**TABLE 1:** Comparison of unrestricted and restricted models.

	*df*	CMIN	*p*	CMIN/df	RMSEA	AGFI	GFI
Unrestricted model	27	95.298	0.00	3.53	0.078	0.879	0.942
Restricted model	28	285.234	0.00	10.187	0.148	0.735	0.868

**TABLE 2:** Assuming the unrestricted model to be correct.

*Model*	*df*	CMIN	*p*	NFI Delta-1	IFI Delta-2	RFI rho-1	TLI rho-2
Restricted model	1	189.936	0.000***	0.149	0.152	0.219	0.227

***p < 0.001.

The degree of freedom (DOF) of the unrestricted model of the potential dimension of “PC - ELE” is 27, and the chi-square value is 95.298 (*p* = 0.000 < 0.05); the DOF of the restricted model is 28, and the chi-square value is 285.234 (*p* = 0.000 < 0.05); and the comparison summary table of the nested model (Table 2) shows the following: The difference of the DOF between the two models is 1 (28–27), and the chi square difference is 285.234 - 95.298 = 189.936; the probability value of significance test of the chi square value difference is 0.000 < 0.05 and reaches the 0.05 significant level, indicating that there are significant differences between the two measurement models of the unrestricted model and the restricted model. Compared with that of the restricted model, the chi-square value of the unrestricted model is significantly smaller, indicating that the difference validity between the two potential dimensions of “PC - learning input” is better.

### Results of the Internal Consistency Reliability Coefficient (ICRC) of Each Questionnaire

Reliability shows whether the results have good internal consistency and stability. The consistency is positively correlated with the reliability of the scale, as shown in Table 3. The ICRC of the PC scale is 0.868, the ICRC of the ELE scale is 0.917, and the ICRC of the PE scale is 0.925. The ICRC of Cronbach’s α of each scale has reached above 0.85, and the ICRC of each dimension of each scale is above 0.7, reaching an acceptable level of reliability.

**TABLE 3:** The results of the internal consistency reliability test of each questionnaire (*n* = 211).

Scale	Sample number (*n*)	Number of topics	Cronbach’s α coefficient	Subscale	Number of topics	Cronbach’s α coefficient
PC questionnaire	211	26	0.868	Se	7	0.864
				Resilience	7	0.861
				Hope	6	0.808
				Optimism	6	0.792
ELE questionnaire	211	17	0.917	Vitality	6	0.801
				Dedication	5	0.806
				Focus	6	0.846
PE questionnaire	211	9	0.925	PE	9	0.925

### Result of CFA of Various Research Variables

Validity refers to the extent to which a thing can be accurately measured. The purpose of CFA is to confirm whether the factors contained in the scale are the same as the constructs originally explored in the questionnaire. The structure of this study was based on the previous theoretical basis, and the hypothesis was also established on the previous research conclusions. The research tools were revised according to the mature scale in previous studies. At this time, the factors of the scale and their items were both fixed, and what the authors want to explore was whether the factor structure model of the scale fitted with the data collected; therefore, this study used AMOS 21.0 to conduct CFA on the scale.

In this study, indicators including χ^2^/df, RFI, TLI, NFI, IFI, GFI, and RMSEA were used as model indicators, and the fitting criteria of each index were χ^2^/df ≤ 3; RFI, TLI, NFI, IFI, and CFI ≥ 0.80, which gets better the closer it is to 1; and RMSEA ≤ 0.1, which gets better the closer it is to 0.

Table 4 shows the χ^2^ test of the PC, that is, χ^2^/df = 1.969 < 3, and the χ^2^ test of the ELE, that is, χ^2^/df = 2.655 < 3. The χ^2^/df of the two scale models is less than 3, so the fitting degree (FD) of the model is good. The χ^2^ test of the PE scale model is χ^2^/df = 2.616 < 3, and the FD is better. The indicators of TLI, NFI, IFI, and CFI of the PC scale model are all above 0.8, and RMSEA is 0.068 < 0.08; therefore, the FD of the PC scale model is very good. These indicators of the PC scale model are all above 0.8, and RMSEA is 0.09 < 0.1; therefore, the FD of the ELE scale model is very good. At the same time, these indicators of the PE scale model are all about 0.8, and RMSEA is 0.09 < 0.10, so the FD of the PE scale model is also very good. Based on the above analysis, it is concluded that the FDs of three scale models are good.

**TABLE 4:** Result of CFA of the scales (*n* = 211).

Scales	χ^2^	*Df*	χ^2^*/df*	TLI	RFI	NFI	IFI	GFI	RMSEA
PC	577.032	293	1.969	0.833	0.810	0.839	0.852	0.823	0.068
ELE	307.929	116	2.655	0.865	0.800	0.830	0.887	0.855	0.08
PE	178.632	27	2.616	0.844	0.822	0.866	0.884	0.84	0.09

### Descriptive Statistics and Correlation Analysis of Research Variables

As shown in Table 5, PC is positively correlated with learning input (*r* = 0.54, *p* < 0.01) and PE (*r* = 0.58, *p* < 0.01), and PE is positively correlated with ELE (*r* = 0.39, *p* < 0.01), which conforms to the theoretical expectation, and the hypothesis through the structural equation model will be further validated.

**TABLE 5:** The descriptive statistics of the research data.

Variables	*M*	*SD*	1	2	3	4	5	6	7
Gender	1.71	0.46	1						
Grade	2.11	0.87	–0.10						
Major	2.20	1.42	0.18**	−0.34*					
Economics	3.93	1.12	0.21**	0.05	0.04	1			
PC	3.56	0.81	0.03	–0.06	0.08	0.70**	1		
ELE	3.14	0.72	–0.01	0.05	–0.05	0.02	0.54**	1	
PE	3.44	0.81	–0.13	0.08	−0.16*	0.08	0.58**	0.39**	1

**p < 0.01; *p < 0.05.

### Difference Test Between the Scores of Each Scale

Through one-way ANOVA and independent-sample *t*-test, there is no significant difference between the variables of each scale, including the differences in gender, grade, professional experience, and family economic status.

#### Gender Differences

Taking gender as the classified variable and PC, ELE, and PE as dependent variables, the independent-sample *t*-test was performed (see Table 6). The results show that the score of two variables in the ELE and PE of males is slightly higher than that of females and that the males’ PC score is lower than that of females, but the total difference is not significant, and the gender differences are not significant on the four variables.

**TABLE 6:** The *t*-test of each scale in gender.

Scales	Gender	*M*	*SD*	*t*	Sig. (two sides)
PC	Male	3.54	0.54	−0.42	0.68
	Female	3.57	0.50		
ELE	Male	3.14	0.79	0.10	0.92
	Female	3.13	0.69		
PE	Male	3.60	0.77	1.95	0.05
	Female	3.36	0.82		

#### Grade Differences

With grade difference as the independent variable and PC, ELE, and PE as dependent variables, a one-way ANOVA was carried out (see Table 7). The results show that the difference of three variables of PC, ELE, and PE is not significant in all grades.

**TABLE 7:** One-way ANOVA of each scale in grade.

Scales	Grade	*M*	*SD*	*F*
PC	Freshmen	3.65	0.48	2.37
	Sophomore	3.44	0.44	
	Junior	3.56	0.56	
ELE	Freshmen	3.11	0.75	0.44
	Sophomore	3.07	0.71	
	Junior	3.18	0.71	
PE	Freshmen	3.53	0.76	0.79
	Sophomore	3.41	0.83	
	Junior	3.37	0.84	

#### Major Differences

Taking the major as an independent variable and PC, ELE, and PE as the dependent variables, a one-way ANOVA is carried out (see Table 8). The results show that there is a significant difference in PE (*F* = 3.13, *p* < 0.01), and the scores of psychology and primary education in PE are significantly higher than those in other majors. There is no significant difference between PC and ELE in major.

**TABLE 8:** One-way ANOVA for each scale in specialty.

Scale	Major	*M*	*SD*	*F*
PC	Psychology	3.65	0.50	2.15
	PE	3.42	0.47	
	Primary education	3.41	0.62	
	Educational technology	3.58	0.54	
	Preschool education	3.58	0.38	
ELE	Psychology	3.15	0.75	0.99
	PE	3.26	0.73	
	Primary education	2.99	0.79	
	Educational technology	3.31	0.71	
	Preschool education	3.01	0.46	
PE	Psychology	3.60	0.82	3.13^∗^
	PE	3.38	0.78	
	Primary education	3.09	0.86	
	Educational technology	3.60	0.62	
	Preschool education	3.26	0.72	

*p < 0.01.

#### Family Economic Situation Difference

Taking the family economic status as an independent variable and PC, ELE, and PE as dependent variables (see Table 9), the results show that PC has a significant difference in family economic status (*F* = 2.41, *p* = 0.05), the PC of the subjects with a monthly income of higher than 6,000 yuan is significantly higher than that of other subjects, and there is no significant difference in the family economic status between the ELE and the PE. The authors speculate that family economic status will have an effect on a person’s PC. Material richness will give people confidence and let people not shrink in the face of setbacks, thus producing positive speculations about the future. In other speculations, a higher family income shows that parents have a higher level of culture, and such parents are more willing to adopt a good way of family upbringing to cultivate the positive quality of their children, so the child will have a higher PC.

**TABLE 9:** One-way ANOVA in working years of each scale.

Scale	Economy	*M*	*SD*	*F*
PC	Less than 1,000	3.41	0.71	2.41*
	1,000–2,000	3.3	0.81	
	2,000–4,000	3.48	0.47	
	4,000–6,000	3.53	0.45	
	Above 6,000	3.67	0.48	
ELE	Less than 1,000	3.06	1.05	0.70
	1,000–2,000	2.89	0.55	
	2,000–4,000	3.25	0.59	
	4,000–6,000	3.10	0.72	
	Above 6,000	3.14	0.77	
PE	Less than 1,000	3.48	0.67	1.00
	1,000–2,000	3.24	0.97	
	2,000–4,000	3.40	0.78	
	4,000–6,000	3.32	0.88	
	Above 6,000	3.56	0.81	

*p < 0.05.

### Research Hypothesis Test

**FIGURE 1:**
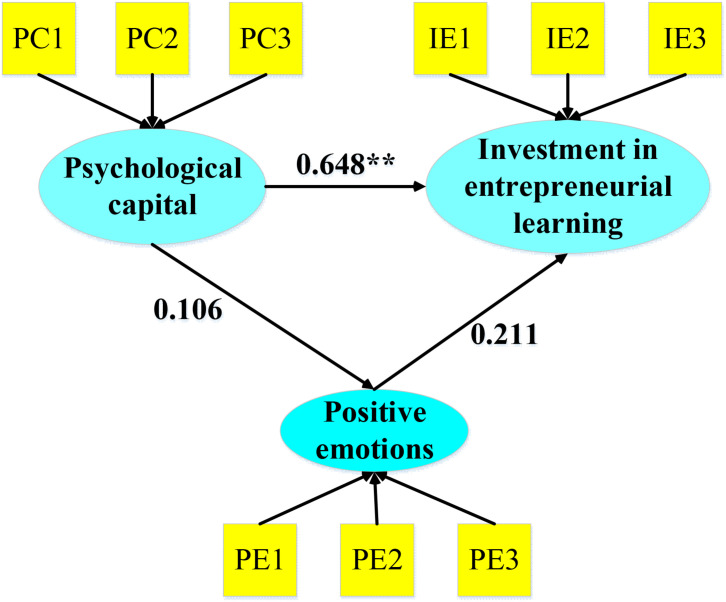
The mediating effect model of PEs in the relationship between PC and ELE. **p < 0.01.

In the study of the hypothetical causal model, the independent variable was college students’ PC, the dependent variable was ELE, and the mediating variable was PE. The DOF of the model was equal to 18, the χ^2^ value of the overall model fitness was 53.505, the significant probability value was 0.000 < 0.001, reaching the 0.05 significant level, and the null hypothesis cannot be accepted, suggesting that the hypothesis model and the sample data cannot fit together. In Figure 1, the direct path coefficient of PC and ELE was 0.648, which was at a significant level (*p* < 0.001); while the indirect path coefficients of PEs added to PC and ELE were 0.106 and 0.211, respectively, which were not significant. Therefore, the PE cannot be used as a mediating variable between the PC of the independent variable and the ELE of the dependent variable.

## Discussion

As a core element, PC focuses on the psychological state and can be obtained through targeted development (Raja et al., 2020). The influencing factors of college students’ ELE based on the PC dimension was analyzed in this study. First, the principal component analysis method was used to test the difference validity of the two variables PC and ELE. It was found that the DOF of the PC–ELE unrestricted model was 27, the chi-square value was 95.298 (*p* < 0.001); the DOF of the model was 28, and the chi-square value was 285.234 (*p* < 0.001), which was basically consistent with the research results of Chipfupa and Wale (2020) and indicated that there was a significant difference between the unrestricted model and the restricted model, PC - learning investment; also, the two potential dimensions of difference were better in validity. In this study, AMOS 21.0 was used to perform a CFA on each scale, and the ICRCs of the PC scale for college students (0.868), ELE scale (0.917), and PE scale (0.925) reached more than 0.85, which fully showed that the internal consistency of each scale was good and that each had high reliability (Jin et al., 2020). In demographic variables, PEs had significant differences at the professional level (*F* = 3.13, *p* < 0.01), and psychological and elementary education scores in PEs were significantly higher than those of other majors, which were different from those found by Terry et al. (2020). The research results were similar, indicating that college students who have been mentally educated for a long time were better at regulating their emotions and maintaining a positive mental state. Moreover, PC had significant differences in family economic status (*F* = 2.41, *p* < 0.01). The PC of subjects with a monthly income of more than 6,000 yuan was significantly higher than that of others. The reason may be that the family’s material wealth conferred a certain degree of confidence in students to a certain extent, and since the parents of students with better family status often had a higher educational level and good family education, these made students have higher PC (Xi et al., 2020). In addition, the structural equation model was also used to analyze the mediating effect of PEs, and it was found that the indirect path coefficients of PEs and psychological learning investment after joining the PC were 0.106 and 0.211, respectively, so there was no significance. This was similar to the results of Raissifar and Akhavan Anvari (2020), which showed that PEs do not play an intermediary role in the relationship between PC and college students’ ELE.

## Conclusion

A single-factor variance and structural equation model were used to analyze the relationship between college students’ PC, ELE, PEs, and differences in demographic variables. The results showed that the PC of college students had a significant PI on ELE, that PC differed significantly in family economic status, and that PEs differed significantly at the professional level. However, the main shortcomings of this study are the selection of mediating variables. Through consulting the literature, the achievement motivation and PE are selected as the mediating variables; however, after the later data processing, it is found that the mediating effect of the two is not significant. On the other hand, due to inadequate sample size, there are inevitable errors; the subjects are selected from students of Qingdao University, which leads to certain limitations. The stability and applicability of the conclusions need to be further validated by selecting more regions and large samples to enhance the reliability of the conclusions. On the basis of this study, the effect mechanism of PC on the result variables of college students’ academic performance, learning performance, and learning satisfaction can be further explored. The results of this study can guide the design and development of relevant teaching practice activities, especially for students who have just entered the university, and the construction of the PC is very necessary. Thus, there was no mediating effect of PEs in the relationship between PC and college students’ ELE.

## Data Availability Statement

The raw data supporting the conclusions of this article will be made available by the authors, without undue reservation, to any qualified researcher.

## Ethics Statement

The studies involving human participants were reviewed and approved by the Ningbo University Ethics Committee. The patients/participants provided their written informed consent to participate in this study. Written informed consent was obtained from the individual(s) for the publication of any potentially identifiable images or data included in this article.

## Author Contributions

All authors listed have made a substantial, direct and intellectual contribution to the work, and approved it for publication.

## Conflict of Interest

The authors declare that the research was conducted in the absence of any commercial or financial relationships that could be construed as a potential conflict of interest.
